# Optimized machine learning model for air quality index prediction in major cities in India

**DOI:** 10.1038/s41598-024-54807-1

**Published:** 2024-03-21

**Authors:** Suresh Kumar Natarajan, Prakash Shanmurthy, Daniel Arockiam, Balamurugan Balusamy, Shitharth Selvarajan

**Affiliations:** 1 School of Computer Science and Engineering, Jain (Deemed-to-be University), Bengaluru, Karnataka India; 2 School of Computer Science and Engineering and Information Science, Presidency University, Bengaluru, Karnataka India; 3 ASET-CSE, Amity University, Gwalior, Madhya Pradesh India; 4 Associate Dean-Student, Shiv Nadar Institution of Eminence, Delhi, India; 5 School of Built Environment, Engineering and Computing, Leeds Beckett University, Leeds, LS1 3HE UK

**Keywords:** Air pollution, Air quality index, Machine learning, Optimization algorithm, Grey-wolf optimization, Decision tree regression, Environmental sciences, Engineering, Computer science

## Abstract

Industrial advancements and utilization of large amount of fossil fuels, vehicle pollution, and other calamities increases the Air Quality Index (AQI) of major cities in a drastic manner. Major cities AQI analysis is essential so that the government can take proper preventive, proactive measures to reduce air pollution. This research incorporates artificial intelligence in AQI prediction based on air pollution data. An optimized machine learning model which combines Grey Wolf Optimization (GWO) with the Decision Tree (DT) algorithm for accurate prediction of AQI in major cities of India. Air quality data available in the Kaggle repository is used for experimentation, and major cities like Delhi, Hyderabad, Kolkata, Bangalore, Visakhapatnam, and Chennai are considered for analysis. The proposed model performance is experimentally verified through metrics like R-Square, RMSE, MSE, MAE, and accuracy. Existing machine learning models, like k-nearest Neighbor, Random Forest regressor, and Support vector regressor, are compared with the proposed model. The proposed model attains better prediction performance compared to traditional machine learning algorithms with maximum accuracy of 88.98% for New Delhi city, 91.49% for Bangalore city, 94.48% for Kolkata, 97.66% for Hyderabad, 95.22% for Chennai and 97.68% for Visakhapatnam city.

## Introduction

Air pollution is one of the serious issues all around the globe. World Health Organization (WHO) reports that around 7 million people affected into numerous diseases because of air pollution. Air pollution increases the chances of asthma, heart issues, skin infections, eye diseases, throat infections, lung cancer, bronchitis diseases, etc., Long-term exposure of air pollutions may increase the chances of premature mortalities. Children might face development issues which includes impaired lung function and cognitive developments. Pregnant women might face issues in their pregnancy journey which includes low birth weight, premature births, etc., In addition to that diseases air pollution introduces serious threat to plant. The large quantity of emissions from vehicles and industries becomes major factor greenhouse effects. Air pollution will introduce serious impacts in economy as it will increase the healthcare cost for both individual as well as the government. Productivity will be affected due to health issues occurred related to air pollution which leads to economic losses for organization and government. The chronic health condition due to air pollution reduces the labor potential which will affect the economy. Huge financial investments are required to mitigate the air pollution which increases the additional expenditure for government. Numerous global forums continuously discussing about the air pollution and its impacts all over the world for the past three, four decades. Compared to developed countries, developing countries strongly affected by air pollution. Developing countries are in need of increasing the employment, revenue and other resources, it opens up the gate for numerous industries which increases the air pollution.

A statistic reports that the global rank of India in air pollution is 8 among 131 countries in 2022. The highly polluted country is Chad a central African country which has AQI of 169. Next place is occupied by Iraq with AQI of 164, followed by Pakistan which has AQI of 159. The fourth place is occupied by Bahrain which has AQI of 157, Bangladesh occupies the fifth place with AQI of 156 and Burkina Faso occupies sixth place with AQI of 155. Kuwait occupies the seventh place with AQI of 151. The average air quality index of India is 144. Another survey reports that, 21 major cities of India are identified as most populated city in the year 2019. From the statistics mentioned above it can be observed that the air pollution in India is need to addressed effectively to secure the environment and human lives. India’s air pollution is mainly due to vehicles, industries, crop burning and domestic cooking. 50% of the pollution in India is mainly due to the industries. Next to industries, vehicles are the second major pollution generating source which occupies 27%. Crop burning in rural and some urban areas leads to 17% of air pollution and remaining pollution occurs due to domestic cooking. More than 2 million Indians faces serious health issues and sometimes die due to this air pollution. The Air act followed in India for pollution prevention and control has poor impacts due to the rule enforcements.

In the beginning of industrial revolution era, fossil fuels such as coal and petroleum are considered as major energy resources. Humans widely utilize this fossil fuels for their needs as it is abundant and can be exploited in numerous procedures. However, air pollution becomes a serious problem to humans as the energy sources are obtained by combusting the fossil fuels. In the process of fossil fuel combustion, numerous gases like nitrogen oxide, carbon dioxide, sulfur dioxide, etc., Due to this emissions, acid rains, greenhouse effects and other metrological disorders occurs in the polluted regions. Other than industries, vehicles in the urban regions causes air pollution. Recently electric vehicles are adopted to reduce the air pollution however it has limitations like less number of charging stations and infrastructures, limitations in batter production, resource extraction and recycling. Moreover, the cost of batteries used in electric vehicles are high. Due to this, electric vehicles couldn’t be implemented full-fledged manner. In rural areas, burning of organic material as fuel affects the quality of breathable air. In the winter season, large amount of stubble is burnt in the fields which increases the air pollution. Sometimes the garbage is thrown into the fire as an alternative approach to clear landfills increases the air pollution rate.

**Figure 1:**
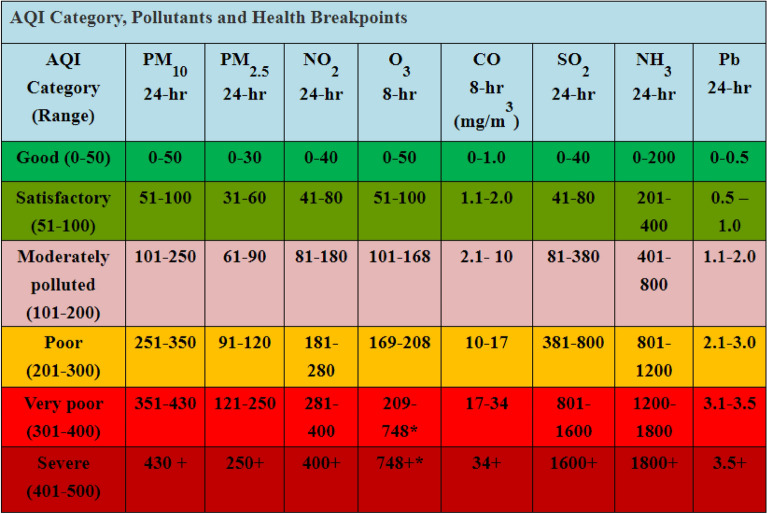
AQI categories for different pollutants.

Due to dangerous pollutant levels and poor quality of air, people in India affected in different ways. For example, in December 2017 Delhi remains closed due to air pollution. The poor quality of breathing air increases the pressure of finding alternate solutions to control the pollution. The poor air quality is generally defined using Air quality index (AQI). The dimensionless factor exhibits air pollution into different quantities. As per the United States Environmental Protection Agency the AQI is categorized into six classes from good to hazardous. The mathematical formulation to calculate the AQI score is given as follows.

1 $$AQI=\frac{{I}_{high }-{I}_{low}}{{C}_{high }-{C}_{low}}\left(C-{I}_{low}\right)+{I}_{low}$$

where pollutant concentration is indicated as $C$, the value less than the normal pollutant concentration is indicated as ${C}_{low}$. The value above the normal pollutant concentration is indicated as ${C}_{high}$. The index breakpoint with respect to ${C}_{low}$ and ${C}_{high}$ is indicated as ${I}_{low}$ and ${I}_{high}$ respectively. A detailed AQI range for different pollutants is presented in Fig. 1 for better understanding.

As given in Fig. 1, the level one AQI is in the range 0–50 and it defines that the quality of air is good and the pollution is minimum in that region. In the second level, the range is fixed into 51–100 and the air quality is mentioned as satisfactory. In the third level, the range is fixed into 101–200 for moderate pollution. In the fourth level the poor pollution status in mentioned with the range of 201–300 AQI score. If the AQI is above 300 and below 400, then it is mentioned as very poor condition. At last, if the AQI is in the range 400–500 then it indicates the severe air pollution in that region.

Prediction of AQI become a hot research topic over a decade and it attracted significant attention all over the world. Various air quality supervision strategies are developed to monitor the pollution level and improve the air quality. Due to this initiatives, the historical monitoring data are widely available and it introduces wide opportunities for researchers in data mining. Analyzing such large amount of data requires better feature processing algorithms and techniques. Thus, numerous models are developed for efficient AQI prediction in the process of air pollution analysis. Earlier air pollution prediction models included statistical linear method for predicting the AQI metrics. However, the statistical linear models have poor estimation results due to time series data variations. Additionally statistical models require complex computations in the prediction process. Statistical method like autoregressive integrated moving average (ARIMA) used for air quality prediction does not provide reliable prediction results for the non-linear data.

To overcome the computation complexity in statistical linear methods, machine learning models are used in recent times. For nonlinear regression forecasting, support vector regression, random forest regression is used. However, due to large data volume this regression models lags in performances. Thus, the complexity can be reduced by selecting optimal features from the dataset in the prediction process. In some methods, back propagation neural networks are employed for prediction analysis. However, those prediction models require long term learning and falls into local minima. The convergence speed is quite low compared to other methods. The features of optimization models are visible in various domains. Numerous optimization algorithms such as dragon fly optimization, alpine skiing optimization are addressed in^1–3^ research work to solve the feature processing limitations of machine learning algorithms. Based on these observations, an objective is defined which can support industries to adjust the production processes. Considering the prediction performances timely adjustments can be made which can reduce the environmental impacts. To attain the desired objective, the contributions are made in this research work are given as follows.

- An optimized regression model for air quality index prediction is proposed using grey wolf optimization algorithm and decision tree regression model.
- The essential optimal features from the dataset are extracted using grey wolf optimization algorithm. The selected optimal features are finally classified using decision tree regression model
- A detailed experimental analysis using benchmark air pollution data is presented and verified the proposed model performance in terms of accuracy, mean absolute error, root mean square error and mean square error.
- A comparative analysis of proposed model metric with existing algorithms for better validation of proposed model.

The following discussion in the article are presented as follows. A detailed literature analysis on different pollution detection and prediction models are presented in “Related work”. Materials, methods, and proposed model are presented in “ Materials and methods ”. Experimental setup, performance metric formulations, performance metric analysis and comparative analysis are presented in detail in “ Final prediction model ”. “ Experimentation, results and analysis ” presents the summary of research work.

## Related work

The recent research works on air quality prediction is considered for literature analysis and the observed summary is presented in this section. Numerous machine learning models are used for AQI prediction^4^. A machine learning based AQI prediction model presented by ^5^ considers environmental monitoring data and metrological measurements for the prediction process. The presented neural network is a non-linear autoregressive model which effectively performs time series prediction and attained robust performances over traditional machine learning based prediction models.

Yang et al.^6^ presented a AQI prediction model which includes a neural network in addition to Gaussian plume model to attain improved prediction performances. The presented adaptive monitoring model measures the AQI level for selected locations and constructs the AQI map. Compared to traditional neural network model, the presented Gaussian plume neural network attained better prediction performance and reduce the power consumption compared to existing monitoring approaches. Gu et al.^7^ reported an air quality prediction model which includes a heuristic recurrent air quality predictor model to predict the pollutants PM 2.5 fine particle matter. Conventional approach predicts AQI using machine learning (ML) and the limitations in such models are overcome by the presented prediction model with minimum time compared to state of art of techniques.

Ameer et al.^8^ presented a real time air pollution monitoring model which includes internet of things (IoT) sensors and machine learning algorithms. Using multiple regression techniques, air pollution is predicted with better accuracy. Experiments confirms that the presented regression model attained minimum RMSE and MAE. Ha et al.^9^ presented a multisensory air pollution data analysis model which fuses the data obtained by wasp mote sensors and humidex data. Further using an extended fractional order Kalman filter the fused data is classified to alert the users in the smart buildings about the air pollution. Timely prediction and alert can be provided by the presented model in an effective manner. Similar IoT based air pollution monitoring system was presented by^10^ for monitoring the air quality of Calgary, Canada. Using mixed edge and cloud-based prediction model the air quality index is predicted with better accuracy and mean absolute error compared to traditional methods.

Chen et al.^11^ presented an auto regressive model for AQI prediction from the data collected by the WSN network. The presented approach initially includes adaptive Kalman filter to fit the data best into the prediction model. Further using auto regressive model the AQI are predicted with better accuracy compared to conventional techniques. However, the obtained accuracy is comparatively low and it can be increased if recent regression models are included in the prediction process.

Lin et al.^12^ presented a four-layer fuzzy neural network model for AQI prediction from historical time series data. The initial fuzzy rules are derived from the time series historical data for better forecasting performances. Using the mean and variance, the membership functions are characterized. The final four-layer fuzzy neural network is obtained based on the fuzzy rules and clusters. Finally, descent backpropagation algorithm, particle swarm optimization, genetic algorithm is used for network training. The major merits of presented approach is its automatic feature extraction and fuzzy rule extraction performances.

An ensemble model presented by^13^ combines multiple machine learning algorithms to develop a better prediction model. conventional machine learning algorithms like support vector machine, k-nearest neighbor, linear regression, and logistic regression are include in the ensemble approach. experimental results confirmed that the ensemble model attained better prediction performances over conventional machine learning methods.

The AQI prediction model presented by^14^ includes support vector regression and LSTM model to classify AQI values. The presented approach initially extracts the mean, MSE and standard deviation from the data using grey level co-occurrence matrix. Further the extracted features are classified using combined approach and exhibit the better performances over conventional machine learning models.

A hybrid model presented by^15^ combines artificial neural network, factor analysis, auto regressive moving average models to attained better feature extraction and prediction performances. Initially the pollutant components are extracted using factor analysis. The extracted features are fed into artificial neural network regression model to analyze the projected rate. The presented hybrid approach attained better prediction performances over machine learning methods in terms of MSE, RMSE and accuracy metrics.

Liu et al.^16^ presented a reliable AQI prediction model which includes initially decomposes the AQI using variational mode decomposition improved by sample entropy. Further using LSTM network is used to produce high quality time series data. Finally using least square support vector machine the features are processed. To improve the prediction performances of support vector machine, the parameters of SVM are optimized by bat algorithm. compared to other hybrid models, the performance of presented optimization algorithm is much better in terms of accuracy for all the classes.

Li et al.^17^ presented a complete ensemble empirical model decomposition and multiscale entropy for AQI prediction. Initially AQI data is decomposed using empirical model decomposition. Further using intrinsic mode function of bald eagle search algorithm, the intrinsic model function components are obtained. Finally, rat swarm optimized kernel ELM is used to attain better prediction performances. Though the presented model attained better performance the computation complexity of presented approach is comparatively high.

Yang et al.^18^ presented a AQI prediction model to measure the quality of Beijing and Taiyuan city using regression model. The presented approach initially includes variational model decomposition model for data decomposition. Further for the residual decomposed components, a second level decomposition is performed. Finally using improved support vector regression, the components are reconstructed with better correlation. Presented approach attained better prediction performances with better MSE and RMSE values.

Maltare et al.^19^ presented a comparative analysis of various machine learning models like SVM, SARIMA and LSTM models for AQI prediction for Ahmedabad city. The presented approach initially removes the redundant data and empty cells in the dataset in the preprocessing stage. Further the preprocessed data is fed into multiple classifiers and their performances are analyzed. Experimental results confirms that support vector machine model better performances over other models.

A dynamic graph neural network based AQI prediction model presented by^20^ includes adaptive edge attributes to attain better prediction performances. The presented approach initially generates bidirected dynamic graph using the model parameters edge attributes. Due to this, adaptive edge information is gained in the prediction process and attained better prediction performances over conventional methods. A machine learning based AQI prediction reported by^21^ includes XGBoost, k-nearest neighbor, decision tree, linear regression and random forest models. Additionally deep learning models like LSTM are also considered for analysis. Experimental results confirm the best performance of XGBoost regression model over conventional learning algorithms in terms of accuracy and R-square metrics.

Numerous hybrid models are evolved in recent times for AQI prediction^22^. Hu et al.^23^ presented a hybrid prediction model which includes multi-scale temporal feature extraction in the initial phase. Further extraction of spatial features is done in the second phase through hierarchy division. The final hybrid model considers the spatial and temporal features and attained better prediction performances over conventional techniques. Wang et al.^24^ presented a AQI prediction which considers the AQI spatial patterns in terms of predominant air pollutant contribution, magnitude in the prediction process. The presented approach considers the spatiotemporal features for prediction process and provided better cross validation, RMSE and R-square values compared to conventional regression models.

A machine learning based AQI prediction model presented by^25^ developed a two-stage feature selection and regression model to attain better prediction performances. The spatial temporal features from the historical air pollution data are extracted in the two-stage feature selection model. Further using regression model, better prediction performances is attained by the presented approach compared to conventional methods. A hybrid machine learning model presented by^7^ includes nonlinear auto regressive moving average model and deep neural network to attain better prediction performances. Additionally, the presented approach performs automatic feature generation and selection which reduces the computation complexity in the prediction process.

Recently deep learning models are widely used for better classification and prediction performances. Liu et al.^26^ presented a prediction model which exploits the historical air quality data to find the AQI. The presented approach overcomes the limitations of conventional seq2seq prediction model by reducing the training speed and by replacing the original RNN with fully connected encoder. Due to this improved performance the training time of the prediction model is greatly reduced compared to conventional methods. A hybrid model presented by^27^ includes multiple deep learning models to attained better prediction performances. Wang et al.^28^ presented a hybrid deep learning model which includes attention gate unit and convolutional neural network for AQI prediction. The initial features are extracted using convolutional neural network and then classified using attention gate unit. Presented approach effectively overcomes the exploding gradient and vanishing gradient issues of recurrent neural network-based prediction model.

Wang et al.^28^ presented a prediction model which includes LSTM, GRU and Temporal CNN for multiple term prediction. Initially a feedback variational mode decomposition algorithm for decomposing the PM2.5 data. Further the high impact factors are extracted using Copula entropy. Further the three prediction models are fed with the features and their performances are evaluated. The results of individual model is finally combined using Gaussian process regression and attained better prediction performances. However, the computation complexity of the presented model is high due to multiple algorithms.

The deep learning based AQI prediction model presented by^29^ includes LSTM and GRU models. The presented approach combines the deep learning models to predict PM2.5 pollutant data. The performance of the presented hybrid model is compared with machine learning models like support vector machine, linear regression, k-nearest neighbor, LSTM and GRU models. Compared to all the presented model attained better MAE value and R-square value.

Kumar et al.^30^ conducted a risk assessment process to measure atmospheric hydrocarbons in India. Using principal component analysis and diagnostic ratio analysis, the major emission sources were identified as biomass and coal in rural areas. Sudarshan et al.^31^ have analyzed the impacts of polycyclic aromatic hydrocarbons in water and residues. Similarly, Ambade et al.^32,33^ presented the impacts of black carbon and hydrocarbons, analyzing pollution sources in detail. The results revealed that paddy residue emitted a higher level of pollutants compared to other fuels. Hussain et al.^34^ reported the contribution of traffic to air pollution in detail through a comprehensive assessment conducted in traffic regions in East India. The experiment reports that fossil fuels are the major source of black carbon emissions, leading to significant health issues in India.

Kumar et al.^35^ presented a machine learning based air pollution prediction model which includes five machine learning algorithms to evaluate the prediction performances. The case study considered six years of air pollution data from 23 Indian cities and analyzes the air quality. The experiments initially balance the data using resampling technique and then classifies using machine learning algorithms. The results confirms that the Gaussian Naïve Bayes outperformed than other machine learning algorithms in predicting the air pollution in major cities.

Rakholia et al.^36^ analyzed the air pollution concentration in Ho Chi Minh City using machine learning model. The presented multivariate model considers the air quality data, and metrological conditions from urban traffic, industrial areas, residential pollutions, and concentration of hazardous gases. Through static and conditional correlation analysis, a feature set for each pollutant was created and developed a global forecasting model based on neural network to attain better forecasting performances. Yuting et al.^37^ presented a AQI forecasting model which includes variational model decomposition model and whale optimization algorithms. Using variational mode decomposition, the original AQI sequence is decomposed. The parameters of VAD are optimized using whale optimization algorithm to attained improved performances. Finally using bidirectional LSTM model, the dynamic characteristics of features are analyzed and attained better prediction performances over conventional techniques. The literature survey explores various approaches and models employed in predicting air quality indices (AQI), focusing on recent advancements in machine learning and deep learning techniques. Numerous studies have addressed the complexity of AQI prediction, acknowledging the significance of accurate forecasting for environmental monitoring and public health. Machine learning models have been extensively utilized for AQI prediction, showcasing promising results. Notably,^38,39^ utilized environmental monitoring data and meteorological measurements^40^ to develop a non-linear autoregressive model, demonstrating robust performance over traditional methods. Hybrid models, combining multiple algorithms, have emerged as effective strategies for AQI prediction. An ensemble models incorporating support vector regression and LSTM have shown improved classification of AQI values^41^. A reliable model employing variational mode decomposition and LSTM networks, achieving superior accuracy compared to conventional methods^42^. Deep learning techniques have also gained traction in AQI prediction due to their ability to handle complex data patterns. An hybrid deep learning model integrating attention gate units and convolutional neural networks, effectively addressing challenges such as gradient issues^43,44^. Similarly, LSTM and GRU models have been combined to predict PM2.5 pollutant data with remarkable accuracy^45^. Moreover, studies have explored the impacts of air pollution sources, including traffic emissions and industrial activities, on AQI levels^46,47^. A risk assessments is conducted to identify major emission sources, analysed air pollution concentrations using multivariate models, highlighting the significance of comprehensive assessments in urban areas^48,49^. In conclusion, the literature survey underscores the significance of advanced modeling techniques in AQI prediction^50–52^, paving the way for more accurate and reliable environmental monitoring systems^53^. Future research should focus on addressing computational complexities and integrating real-time data for enhanced forecasting capabilities.

## Materials and methods

### Materials

#### Dataset

**Figure 2:**
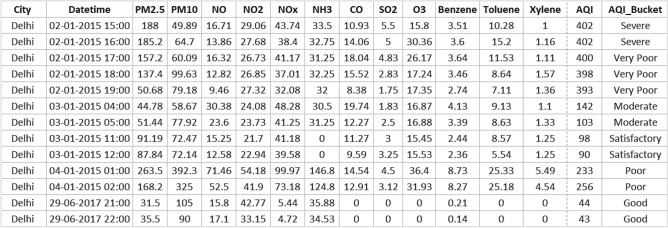
Sample data in air quality dataset for Delhi City.

**Figure 3:**
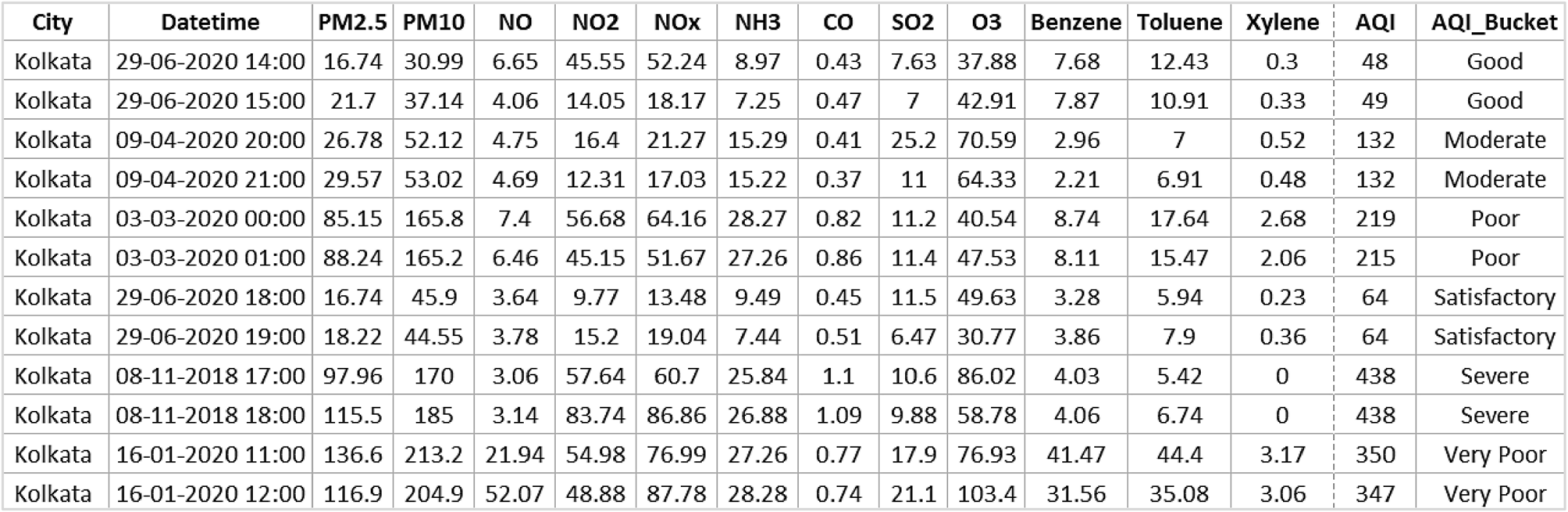
Sample data in air quality dataset for Kolkata city.

The dataset used in the proposed model evaluation is a publicly available Air Quality Data in India (2015–2020) from Kaggle repository^54^. The dataset includes air quality data and air quality index (AQI) data for hourly and daily levels of various stations across major cities of India. The selected cities are Ahmedabad, Aizawl, Amaravati, Amritsar, Bengaluru, Bhopal, Braj Rajnagar, Chandigarh, Chennai, Coimbatore, Delhi, Ernakulam, Gurugram, Guwahati, Hyderabad, Jaipur, Jorapokhar, Kochi, Kolkata, Lucknow, Mumbai, Patna, Shillong, Talcher, Thiruvananthapuram, Visakhapatnam. The attributes in the data for each city are data, month, year, PM_2.5_, PM_10_, NO, NO_2_, NO_x_, NH_3_, CO, SO_2_, O_3_, Benzene, Toluene, AQI, and AQI_Bucket. The AQI bucket is categorized the AQI into six categories as good, satisfactory, moderate, poor, very poor, and severe. Few samples from the dataset for major cities Delhi and Kolkata are presented in Figs. 2 and 3.

#### Preprocessing

**Figure 4:**
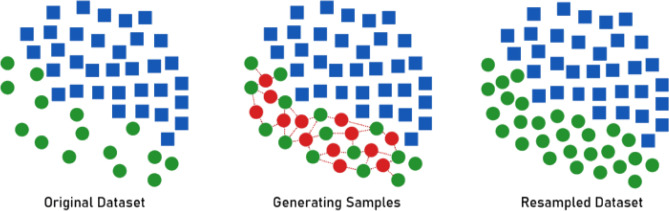
Data balancing using SMOTE.

The dataset used in the proposed model experimentation has null values. Thus, it requires data cleaning. In this process, the null values are removed from the dataset and then the remaining data are used for experimentation. However, AQI bucket has six categories and for the major cities selected, the data becomes imbalanced after null removal. Thus, to balance the data, synthetic minority oversampling (SMOTE) is employed. Using SMOTE the imbalanced dataset is converted into balanced dataset. SMOTE performs oversampling of data and the inadequate data are supplemented with additional data. Generally SMOTE finds a feature vector and its closet neighbor and obtains the difference values. Then the difference is multiplied with a random number between zero and one to find the new data point. This process is repeated for all the feature vectors. Compared to other data balancing algorithms, SMOTE provides better balancing by generating synthetic data points. The major benefit of smote over other data balancing technique is SMOTE does not produce duplicate data points. It produces artificial data points with marginal difference from actual data points. A simple illustration that describes the sample generation and resampling performed in SMOTE is depicted in Fig. 4.

### Methods

#### Grey wolf optimization

In the proposed model after data balancing, nature inspired grey wolf optimization is incorporated to select the optimal features from the dataset. Grey wolf optimization (GWO) is formulated based on the hunting strategy of grey wolf. Compared to other optimization algorithms, GWO can effectively overcome the local optimal trap and provides better exploration characteristics in search space. Generally grey wolf follows leadership hierarchy for the hunting process. wolf groups are mainly categorized into four such as alpha, beta, delta and omega. To represent the leadership hierarchy, alpha the male is considered as a leader for the group. All the decisions are made by alpha in the hunting process, sleeping time, location etc., The beta helps the alpha in the decision-making process. If the alpha is not present or died then the beta will become alpha. Omega wolves occupies last positions and these wolves will always work under the alpha and beta wolfs. Generally, omega wolves are considered as a group and it will work mainly in the hunting process based on the decisions of alpha. The wolves which do not comes under alpha, beta and omega are considered as delta wolves. These delta wolves are dominant to omega volves and comes under alpha and beta. Generally, care takers, scouts, elders, sentinel are coming under this delta group.

**Figure 5:**
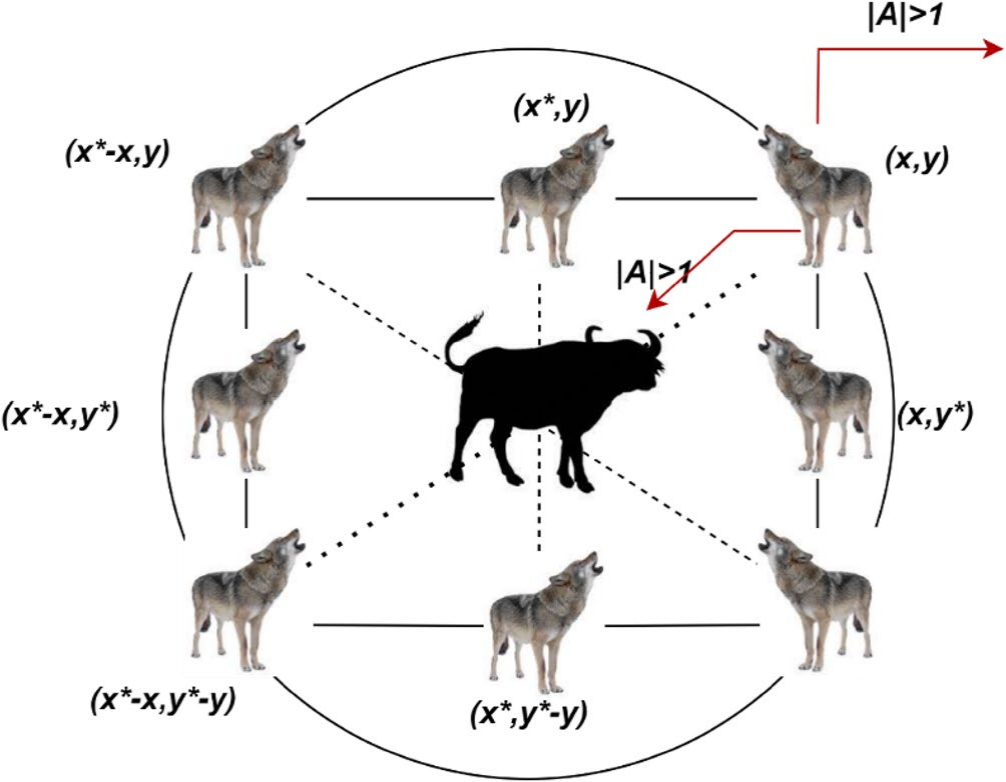
Grey wolf optimization algorithm.

In Fig. 5, the hunting strategy of grey wolves are defined as search for prey, prey encircling and attacking. In the first phase, search for prey, wolves track, chase and approach the prey. In the encircling process, wolves encircle and harassing the prey. In the final attacking phase, direct attack is performed to capture the prey. While formulating the optimization model, the first fittest solution is considered from alpha ($\alpha$), the second and third best fittest solution is considered from beta ($\beta$) and delta ($\delta$) wolves. Consider $w$ wolves which works based on the comments of $\alpha$, $\beta$ and $\delta$ wolves. In the hunting process the prey encircling process is mathematically formulated as

2 $$\overrightarrow{k}=\left|\overrightarrow{d}.\overrightarrow{{i}_{p}}\left(t\right)-\overrightarrow{I}(t)\right|$$

3 $$\overrightarrow{I}\left(t+1\right)=\overrightarrow{{I}_{p}}\left(t\right)-\overrightarrow{A}.\overrightarrow{k}$$

where the coefficient vectors are indicated as $\overrightarrow{d}$ and $\overrightarrow{A}$, the prey position vector is indicated as $\overrightarrow{{I}_{p}}$ and the grey wolf position vector is indicated as $\overrightarrow{I}$. The current iteration is indicated as $t$. The coefficient vectors $\overrightarrow{d}$ and $\overrightarrow{A}$ can be calculated as follows.

4 $$\overrightarrow{d}=2.\overrightarrow{{r}_{2}}$$

5 $$\overrightarrow{A}=2\overrightarrow{a}.\overrightarrow{{r}_{1}}-\overrightarrow{a}$$

where the random vectors are indicated as $\overrightarrow{{r}_{1}}$ and $\overrightarrow{{r}_{2}}$ and its range is given as [0, 1]. During the iteration, the component $\overrightarrow{a}$ is linearly decreased from 2 to 0. Figure 4 depicts a simple illustration of grey wolf optimization prey encircling process.

In the prey hunting phase, the positions of omega are updated frequently based on the first best three solutions obtained by the alpha, beta and delta wolves. The update process is mathematically formulated as follows.

6 $$\overrightarrow{{k}_{\alpha }}=\left|\overrightarrow{{d}_{1}}.\overrightarrow{{I}_{\alpha }}-\overrightarrow{I}\right|$$

7 $$\overrightarrow{{k}_{\beta }}=\left|\overrightarrow{{d}_{1}}.\overrightarrow{{I}_{\beta }}-\overrightarrow{I}\right|$$

8 $$\overrightarrow{{k}_{\delta }}=\left|\overrightarrow{{d}_{1}}.\overrightarrow{{I}_{\delta }}-\overrightarrow{I}\right|$$

9 $$\overrightarrow{{I}_{1}}=\overrightarrow{{I}_{\alpha }}-\overrightarrow{{A}_{1}}.\left( \overrightarrow{{k}_{\alpha }}\right)$$

10 $$\overrightarrow{{I}_{2}}=\overrightarrow{{I}_{\beta }}-\overrightarrow{{A}_{2}}.\left( \overrightarrow{{k}_{\beta }}\right)$$

11 $$\overrightarrow{{I}_{3}}=\overrightarrow{{I}_{\delta }}-\overrightarrow{{A}_{3}}.\left( \overrightarrow{{k}_{\delta }}\right)$$

12 $$\overrightarrow{I} \left(t+1\right)=\frac{{I}_{1}+{I}_{2}+{I}_{3}}{3}$$

**Figure 6:**
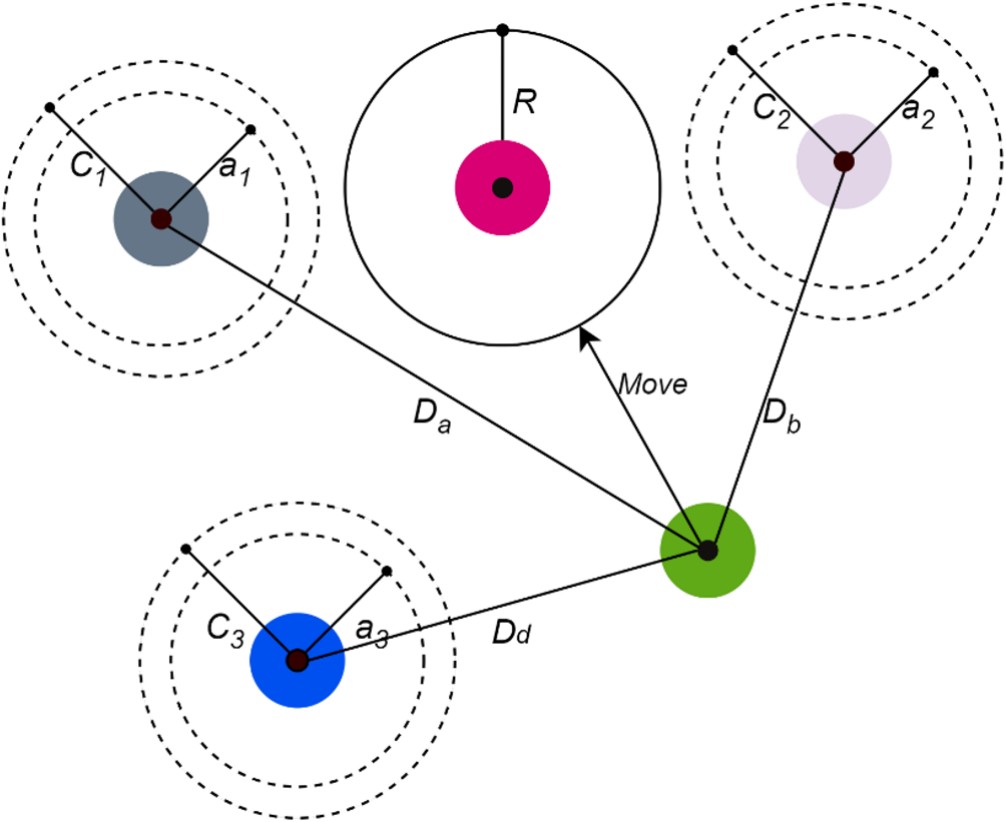
Position update in grey wolf optimization algorithm.

The position update process in grey wolf optimization is illustrated in Fig. 6. The alpha, beta and delta estimate the prey position and other wolves update their position stochastically around the prey. In the final prey attacking phase, the wolves finish the hunt when the prey stops its movement. mathematically the process of wolves approaching the prey is formulated considering the coefficient vector $\overrightarrow{A}$ and $\overrightarrow{a}$. If the wolf approach towards the prey in the hunting process, then $\overrightarrow{a}$ decrease and due to this there is a fluctuation in coefficient vector $\overrightarrow{A}$. If the value of $\left|A\right|<1$ then wolves converge towards the prey and hunt it otherwise it moves away from the prey and start searching another prey.

Based on the solutions of the optimization algorithm, the optimal features are extracted from the dataset and then used to predict the air quality using decision regression model. The learning characteristics of decision tree are used to solve classification and regression problems. The reason for selecting decision tree is its simple tree structure which allow to trace decision paths and realize the logic behind predictions. Compared to linear models, the performance of decision tree regression will be better. The feature importance in decision tree regression will be better thus its prediction performance will be better compared to other prediction procedures.

#### Decision tree regression

The decision tree algorithm is a nonparametric model which is widely used to predict qualitative and quantitative variables. Data responses can be predicted using decision trees. The tree like structure in the decision tree provides data classification and regression by illustrating the direct and indirect correlations between independent and target variables. In the tree structure, the upper branches have greater prediction factors for the related class. The classification in the decision tree provides nominal responses while the regression provides numeric responses. In the prediction process, the decisions are obtained from the root node to leaf node. In general, the leaf node contains the responses. The relationship between feature vectors and displacement vectors are mapping in the regression model using a regressor ${R}_{l}$. Then using the regressor the displacements in the features vectors are predicted. Finally, the predicted values are considered as an optimal solution for the given problem. In the proposed model regression is used instead of classification to obtain better results while evaluating region of interest.

### Final prediction model

The final prediction model using grey wolf optimization and decision tree regression is presented in Fig. 6. In the initial preprocessing step, data cleaning and null value removal is performed. From the pre-processed data, the optimal features are extracted using grey wolf optimization algorithm. The extracted optimal features are analyzed using decision tree regression model for predict the AQI. The proposed model is initially trained with the dataset and then tested using sample test data. For this process, the entire dataset is divided into 80:20 for training and testing. The final testing performances are measured using performance metrics like mean square error (MSE), Mean absolute error (MAE), Root mean square error (RMSE), and accuracy metrics.

The DT design follows structures similar to a tree like root, branches, and leaves. For instance, AQI as response (Y) is predicted based on multiple predictors (X) that provide categorical AQI as daily or monthly. During the training period, AQI as most available values is given to train the model for recognizing specific features and weather and climate variations. To note, this AQI value also can be any air pollutant to estimate air quality based on the available and accurate observations are there.

**Figure 7:**
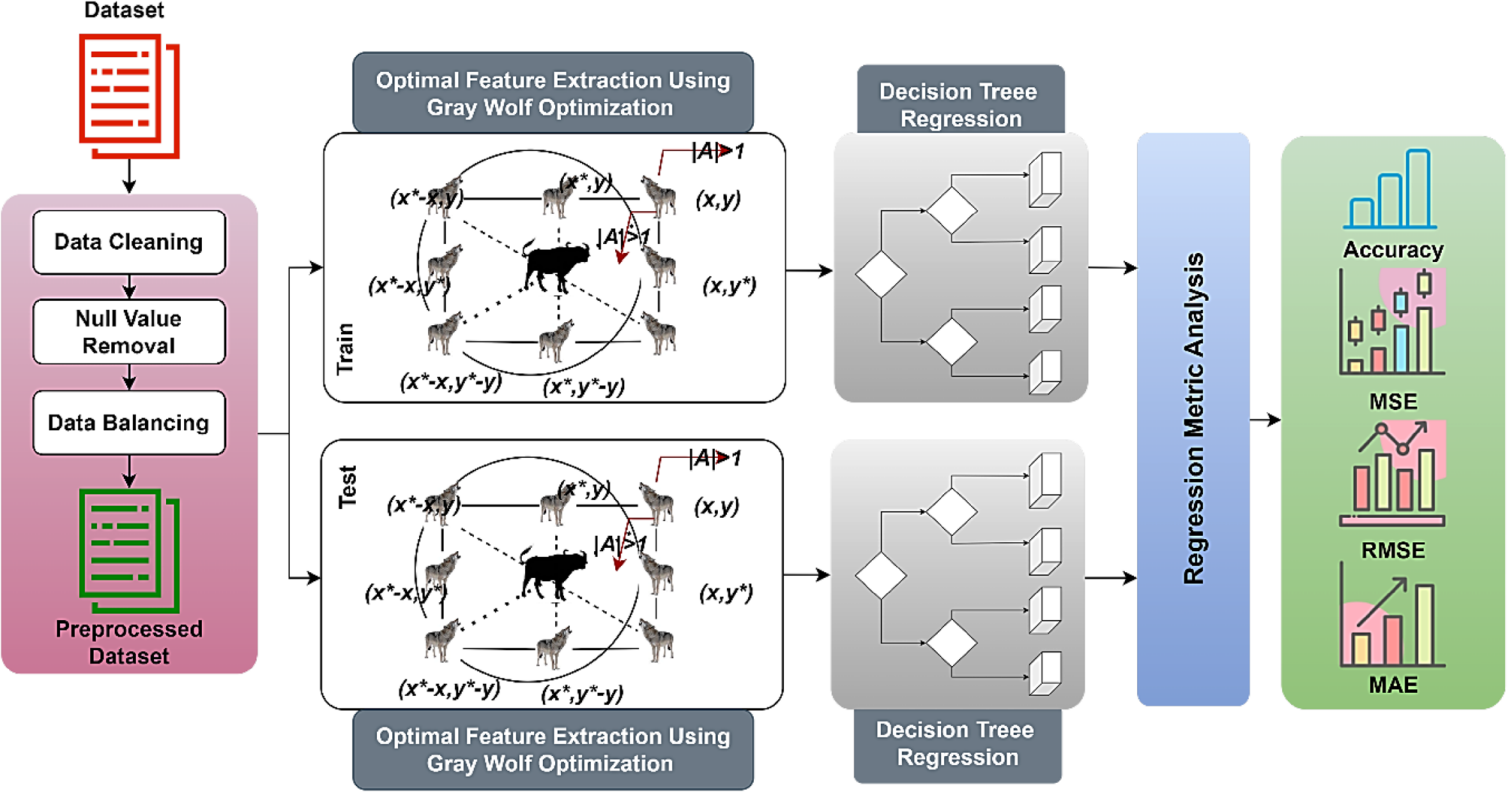
Proposed prediction model.

The final decision tree regression structure has root, branches, and leaves to measure the AQI or the given features. The prediction is performed based on the AQI categorial values in the dataset. In the training process, the available values are used to train the model so that specific features can be learned by the system. In the testing process, decision tree regression test AQI in internal nodes, roots, branches and provided the final prediction results. The proposed prediction model is given in Fig. 7.

In the training process of regression model, the tree ${R}_{l}$ is constructed using landmark $l$. The optimization criteria and stopping criteria must be defined to determine the tree growth. By selecting or pruning appropriate parameters, the decision tree can be developed. The optimization parameter which is used in the regression tree is to minimize the mean squared error in prediction process. Data splitting is a major process in creating decision trees. Basically, four steps are performed to split the node in the decision tree. Initially for each observations the weighted MSE of the responses is computed as follows.

13 $${\varepsilon }_{t}=\sum_{j\in T}\frac{{\left({d}_{j}-{\overline{d} }_{t}\right)}^{2}}{N}\mathrm{ for }j=\mathrm{1,2},\dots .,N$$

where $d$ indicates the displacements or responses in the node $t$. The sample size is indicated as $N$ and set of observation indices are represented as $T$. Further the node observation probability is calculated which is mathematically formulated as

14 $$P\left(T\right)=\sum_{j\in T}{\omega }_{j}$$

where weight is represented as ${\omega }_{j}$. In the proposed work the weight factor is defined based on the sample size N as ${\omega }_{j}=1/N$. Further the observed elements are arranged in ascending order. In order to stop the node splitting in decision tree, two rules are generally followed. In the first rule, if the observed node MSE is lesser than te MSE of entire data multiplied by tolerance on quadratic error per node. Secondly if the decision tree reaches the maximum setting values for regression tree, then node splitting can be stopped. In the prediction process, the response for new data can be predicted easily after creating a regression tree. based on the regression tree rules, the specific attributes are selected by the nodes from the new observation and reach the leaf. Step by step it stores the mean displacement. From the stored value and actual value, the difference can be obtained as prediction results in AQI prediction process.

## Experimentation, results and analysis

### Experimental setup

The proposed model simulation analysis utilizes benchmark Air Quality Data in India (2015–2020) from Kaggle repository. The air quality index of 26 major cities in India are included in the dataset. Among all six major cities like New Delhi, Kolkata, Hyderabad, Bangalore, Chennai, and Visakhapatnam are considered for experimentation. The cities are selected based on the AQI bucket given in the dataset which ranges from good, moderate, poor, satisfactory, sever and very poor. The data for these major cities are exported into a csv file and analyzed the pollution levels. The initial optimal features are extracted using optimization model and then classified using decision tree classifier. All the experimentations are performed in python tool and the essential library functions are included for optimization model and classifier models. The hyperparameters used in the experimentation are listed in Table 1.

**Table 1:** Details of hyperparameters used in the experimentation.

S. no.	Algorithm	Parameter	Range/type
1	GWO	Population size	30
2		Γ	10
3		Σ	0.3
4		Error tolerance	1e−3
5	Decision tree	Number of iterations	1000
6		Maximum number of branches	2
7		Number of interval bins	20
8		Maximum depth	10
9		Minimum leaf size	5

### Performance metrics

The proposed model performances are evaluated using different metrics like R-Square, Mean Square Error (MSE), Mean Absolute Error (MAE), Root Mean Square Error (RMSE), and accuracy. The essential formulations for the performance metrics are given as below.

15 $$R-Square=\frac{{SS}_{regr}}{{SS}_{tt}}$$

where ${SS}_{regr}$ indicates the regression sum of squares, ${SS}_{tt}$ indicates overall sum of squares.

The second metrics used for proposed model evaluation is Mean Square Error (MSE). It is the measure which is used to define how closely the results resembles the data points. It is advisable that the MSE has to be minimum. If MSE is equal to zero or near to zero then the model is considered as a perfect model. The mathematical expression of MSE includes observed values, predicted values and number of observations.

16 $$MSE=\sum_{i=1}^{n}\frac{{\left({x}_{i}-{\widehat{x}}_{i}\right)}^{2}}{n}$$

where the observed values are indicated as ${x}_{i}$, the predicted values are indicated as ${\widehat{x}}_{i}$ and the number of observations is indicated as $n$.

The next metric used in the proposed model evaluation is Root Mean Square Error (RMSE) which is used to describe how the data densely distributed along the best fit line. Mathematically RMSE is formulated as

17 $$RMSE= \sqrt{\sum_{i=1}^{n}\frac{{\left({x}_{i}-{\widehat{x}}_{i}\right)}^{2}}{m}}$$

where the observed values are indicated as ${x}_{i}$, the predicted values are indicated as ${\widehat{x}}_{i}$ and the number of observations is indicated as $n$.

The next metric used for performance evaluation is mean absolute error (MAE). It is used to evaluate the absolute distance between the prediction and observed results. Mathematically MAE is expressed as

18 $$MAE=\frac{1}{m}\sum_{i=1}^{n}\left|{x}_{i}-x\right|$$

where absolute error is indicated as $\left|{x}_{i}-x\right|$ and the number of errors is indicated as $n$.

The final metric used for the proposed model evaluation is accuracy which defines how the proposed model identifies the relations in the dataset. In the regression model accuracy is measured using mean absolute error which is formulated as follows.

19 $$Accuracy= \left(1-MAE\right)*100$$

## Results

### Preprocessing and data balancing

The proposed model preprocessing steps includes, data cleaning, null value removal, and data balancing. Initially in the data cleaning process, the attribute xylene was removed from the data as its values are empty for most of the time period. After that in null value removal, the blanks in other attributed are removed so that the final data will have all the values for all the attributes. The dataset AQI are categorized into six different classes such as Severe, Very Poor, Poor, Good, Satisfactory and Moderate. However, after null value removal the dataset is imbalanced. Instead of using imbalanced dataset, the dataset is balanced by using synthetic minority oversampling technique (SMOTE).

**Figure 8:**
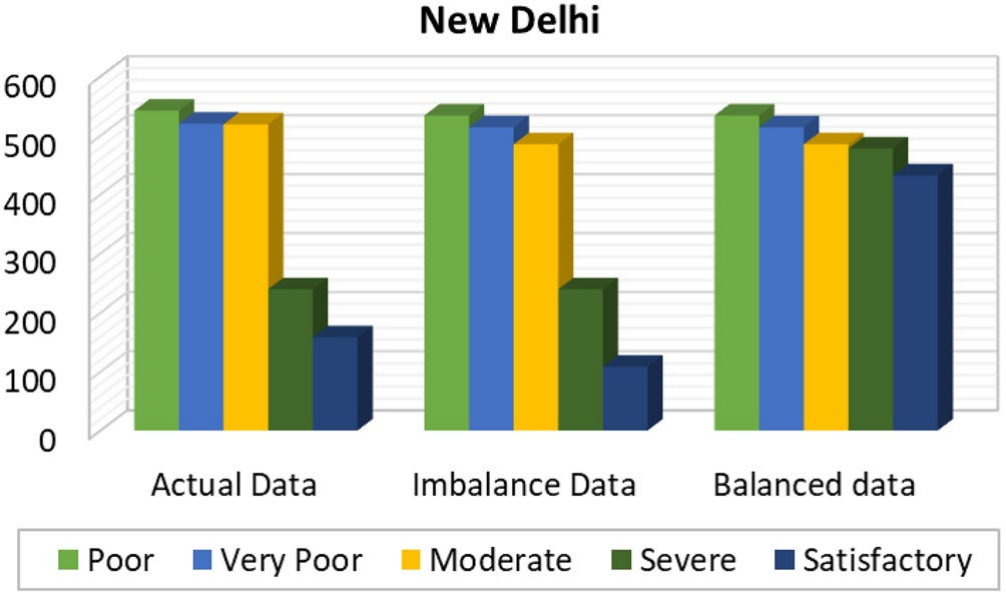
Details of New Delhi city before and after preprocessing and data balancing.

**Figure 9:**
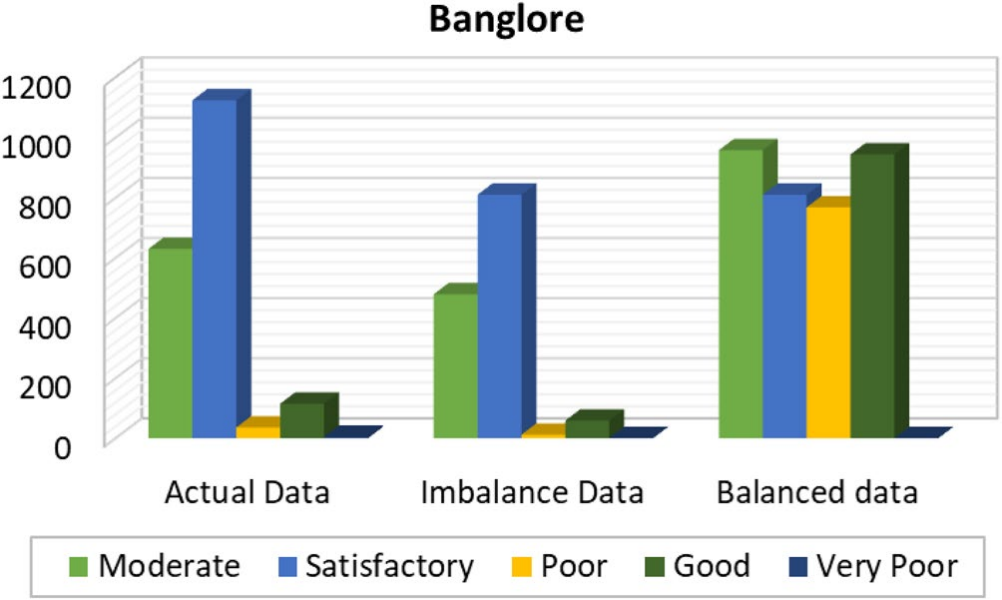
Details of Bangalore city before and after preprocessing and data balancing.

**Figure 10:**
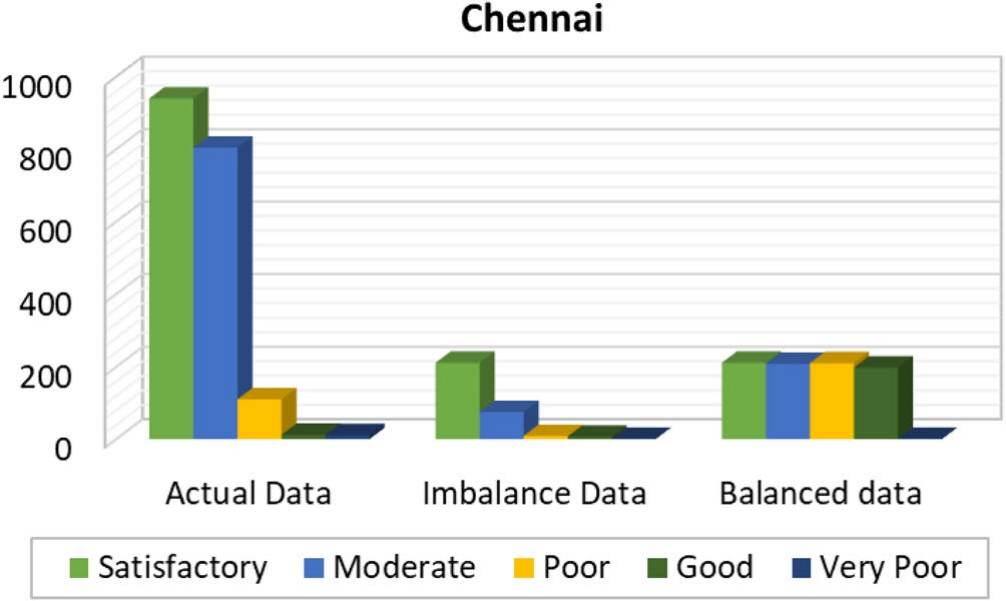
Details of Chennai city before and after preprocessing and data balancing.

**Figure 11:**
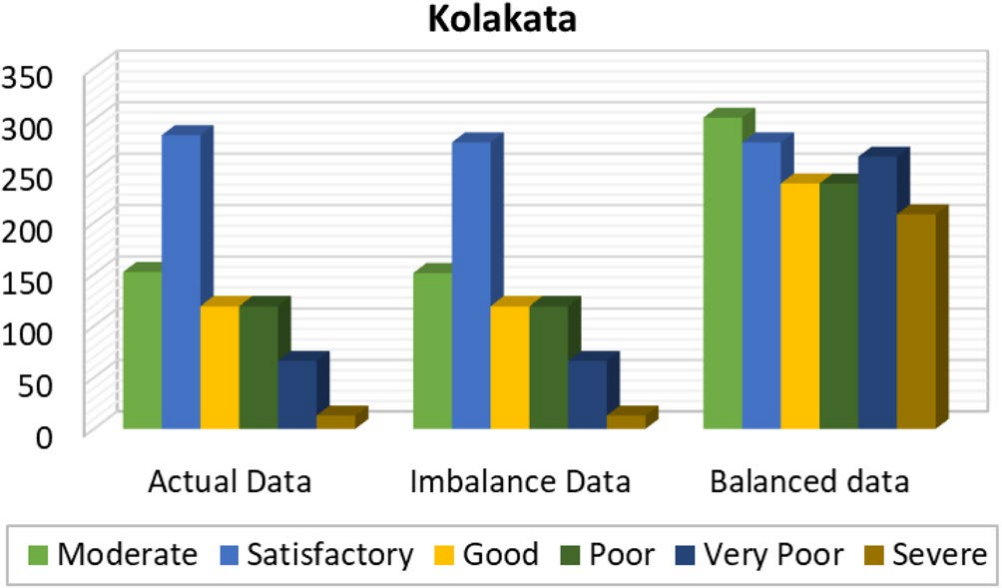
Details of Kolkata city before and after preprocessing and data balancing.

**Figure 12:**
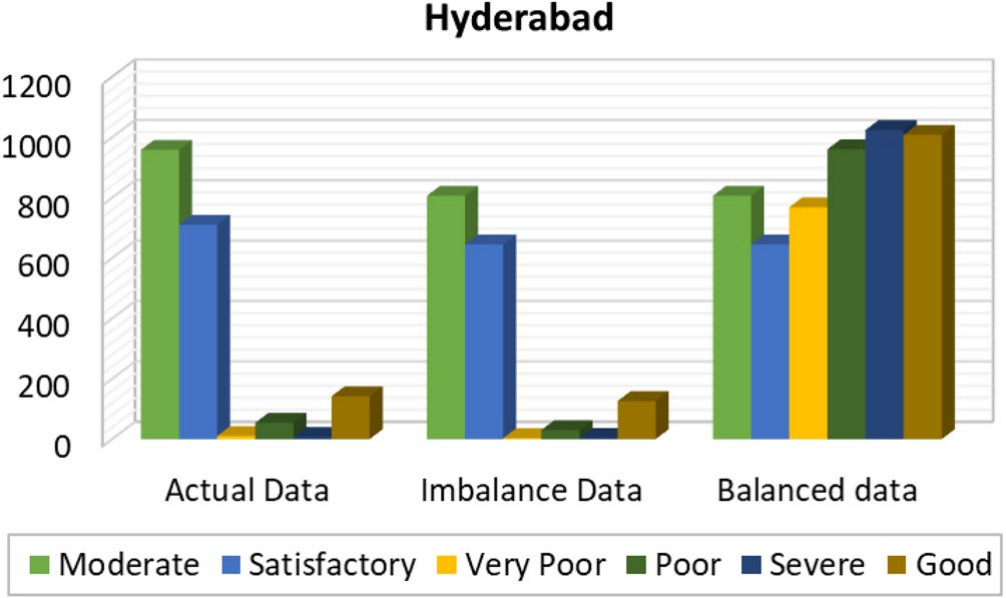
Details of Hyderabad city before and after preprocessing and data balancing.

**Figure 13:**
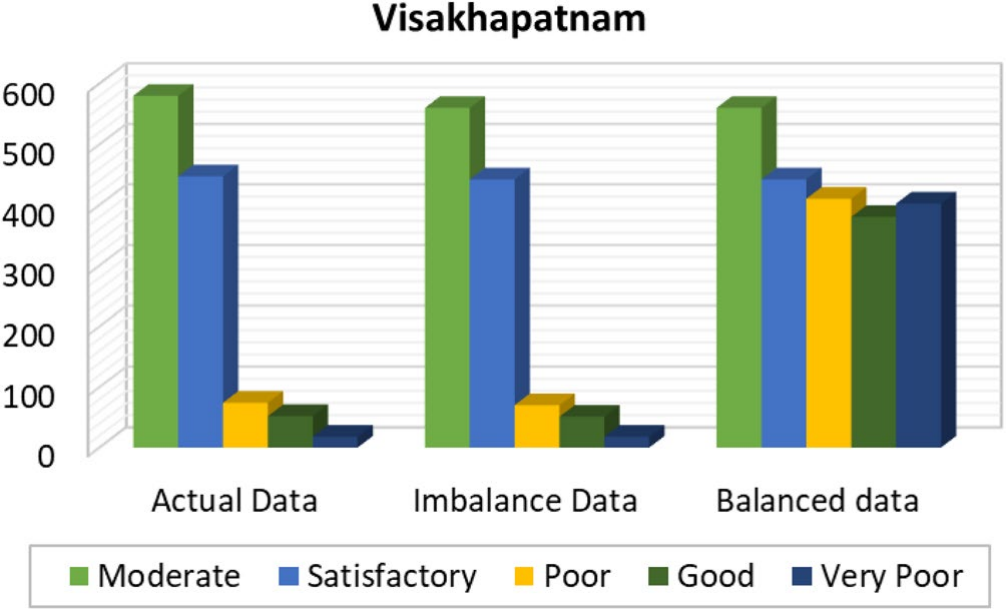
Details of Visakhapatnam city before and after preprocessing and data balancing.

Figures 8, 9, 10, 11, 12 and 13 provides the details for proposed model preprocessing result before and after data balancing for New Delhi, Bangalore, Chennai, Kolkata, Hyderabad, and Visakhapatnam respectively. The first column indicates the different labels in the dataset, the second indicates the actual data for the respective labels. After removing the blanks, the data count is indicated in third column. This data is an imbalanced data and it is balanced using SMOTE. The last column indicates the final balanced data obtained in the proposed model preprocessing step. Further the optimal features are extracted and used to predict the AQI for different cities.

### Performance metrics analysis

The proposed model includes performance metrics like R-Square, Mean Square Error (MSE), Mean Absolute Error (MAE), Root Mean Square Error (RMSE), and accuracy. The performance metrics are evaluated initially for the imbalanced dataset and the evaluated for the balanced dataset. Table 2, 3, 4, 5, 6 and 7 depicts the details of performance metrics for cities New Delhi, Bangalore, Chennai, Kolkata, Hyderabad, and Visakhapatnam respectively.

**Table 2:** Performance metrics of proposed model for New Delhi City.

City: New Delhi			
S. no.	Performance metrics	Imbalanced dataset	Balanced dataset
1	R-square	0.9384	0.9485
2	MSE	0.0764	0.0722
3	RMSE	0.2484	0.2263
4	MAE	0.1534	0.1102
5	Accuracy	84.658	88.984

**Table 3:** Performance metrics of proposed model for Bangalore City.

City: Bangalore			
S. no.	Performance metrics	Imbalanced dataset	Balanced dataset
1	R-square	0.7298	0.7466
2	MSE	0.3054	0.2865
3	RMSE	0.5265	0.5224
4	MAE	0.2874	0.0851
5	Accuracy	71.265	91.486

**Table 4:** Performance metrics of proposed model for Chennai City.

City: Chennai			
S. no.	Performance metrics	Imbalanced dataset	Balanced dataset
1	R-square	0.874	0.886
2	MSE	0.1265	0.1224
3	RMSE	0.1448	0.1365
4	MAE	0.1951	0.0478
5	Accuracy	80.486	95.221

**Table 5:** Performance metrics of proposed model for Kolkata City.

City: Kolkata			
S. no	Performance metrics	Imbalanced dataset	Balanced dataset
1	R-square	0.9868	0.9874
2	MSE	0.0184	0.0178
3	RMSE	0.1304	0.1218
4	MAE	0.0735	0.0552
5	Accuracy	92.65	94.48

**Table 6:** Performance metrics of proposed model for Hyderabad City.

City: Hyderabad			
S. no.	Performance metrics	Imbalanced dataset	Balanced dataset
1	R-square	0.88	0.898
2	MSE	0.1348	0.1322
3	RMSE	0.3247	0.3126
4	MAE	0.0732	0.0234
5	Accuracy	92.684	97.662

**Table 7:** Performance metrics of proposed model for Visakhapatnam City.

City: Visakhapatnam			
S. no.	Performance metrics	Imbalanced dataset	Balanced dataset
1	R-square	0.898	0.9024
2	MSE	0.1486	0.1421
3	RMSE	0.3148	0.3068
4	MAE	0.0716	0.0232
5	Accuracy	92.84	97.68

**Figure 14:**
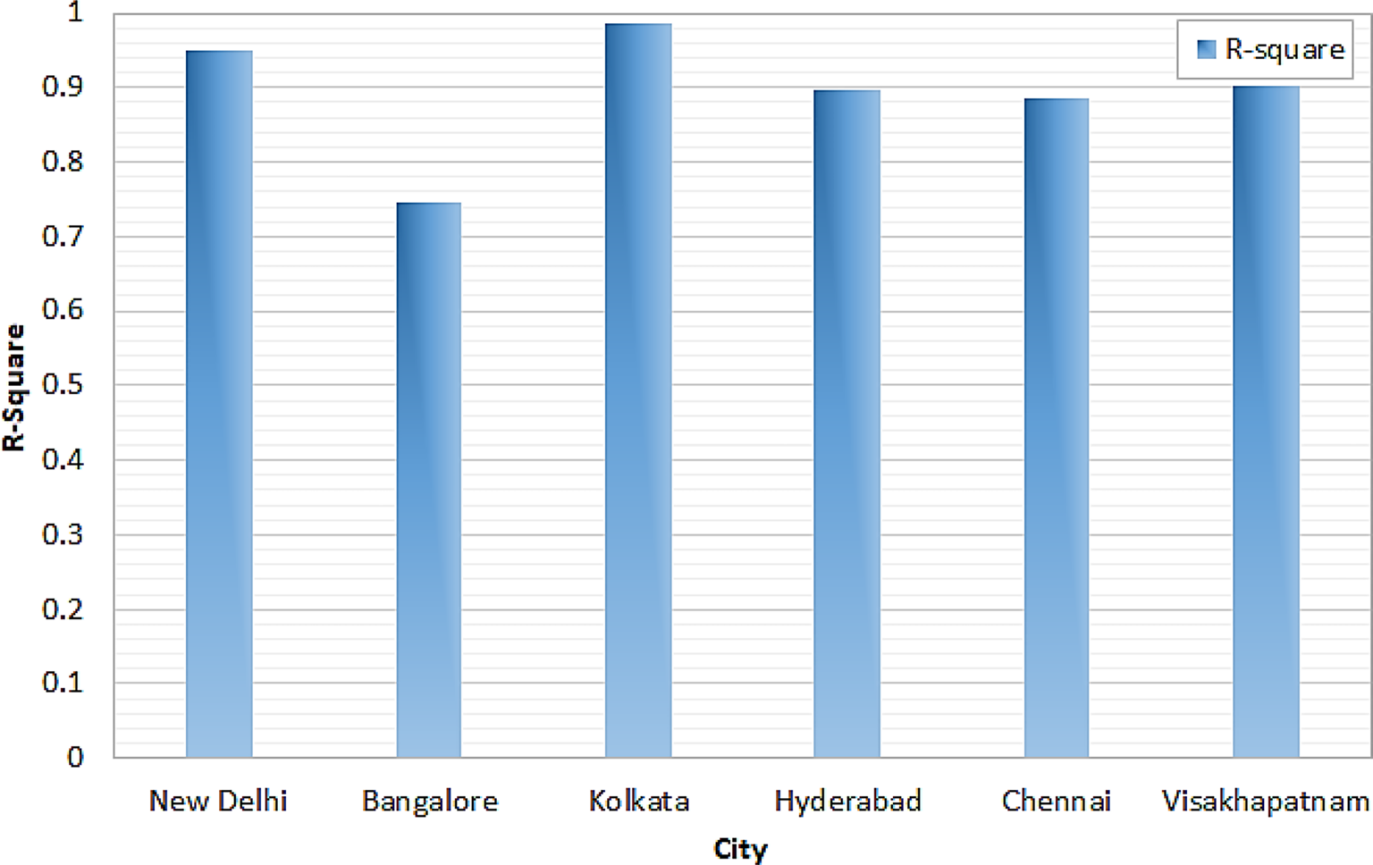
R-square analysis.

The R-Square values obtained by the proposed model for six major cities using balanced dataset is depicted in Fig. 14. It can be observed that, Kolkata has the maximum R-Square value as 0.9874. Next to Kolkata, New Delhi attains 0.9485 as R-square value. The cities like Hyderabad, Chennai and Visakhapatnam have R-square value of 0.898, 0.886 and 0.9024 respectively. The least square was attained for the Bangalore city which has 0.7466 as R-square value.

**Figure 15:**
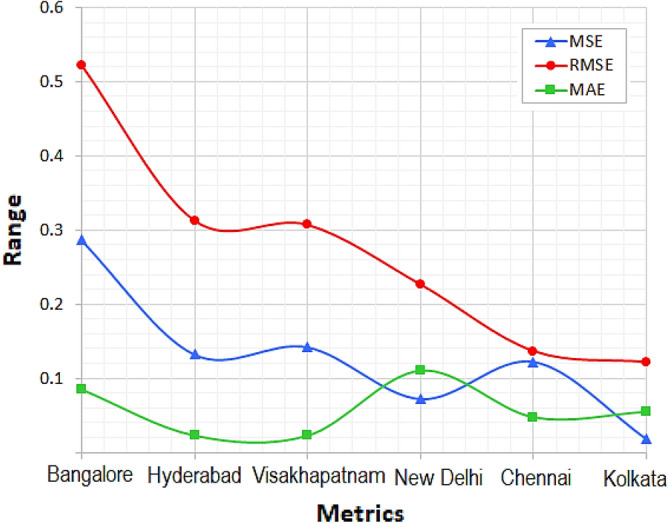
Error analysis.

Figure 15 depicts the error analysis which includes MSE, RMSE and MAE values attained by the proposed model for six cities. Among all Bangalore has maximum MSE, RMSE and MAE values as 0.2865, 0.5224 and 0.0851 respectively. Next to Bangalore, Hyderabad has higher error values as 0.1322, 0.3126, and 0.0234 for MSE, RMSE and MAE respectively. Visakhapatnam has MSE, RMSE and MAE values as 0.1421, 0.3068 and 0.0232 respectively. New Delhi has MSE, RMSE and MAE values as 0.0722, 0.2263 and 0.1102 respectively. Chennai city has 0.1224, 0.1365 and 0.0478 vales for MSE, RMSE and MAE respectively. The least error values are obtained for Kolkata city as 0.0178, 0.1218 and 0.0552 for MSE, RMSE, and MAE respectively.

**Figure 16:**
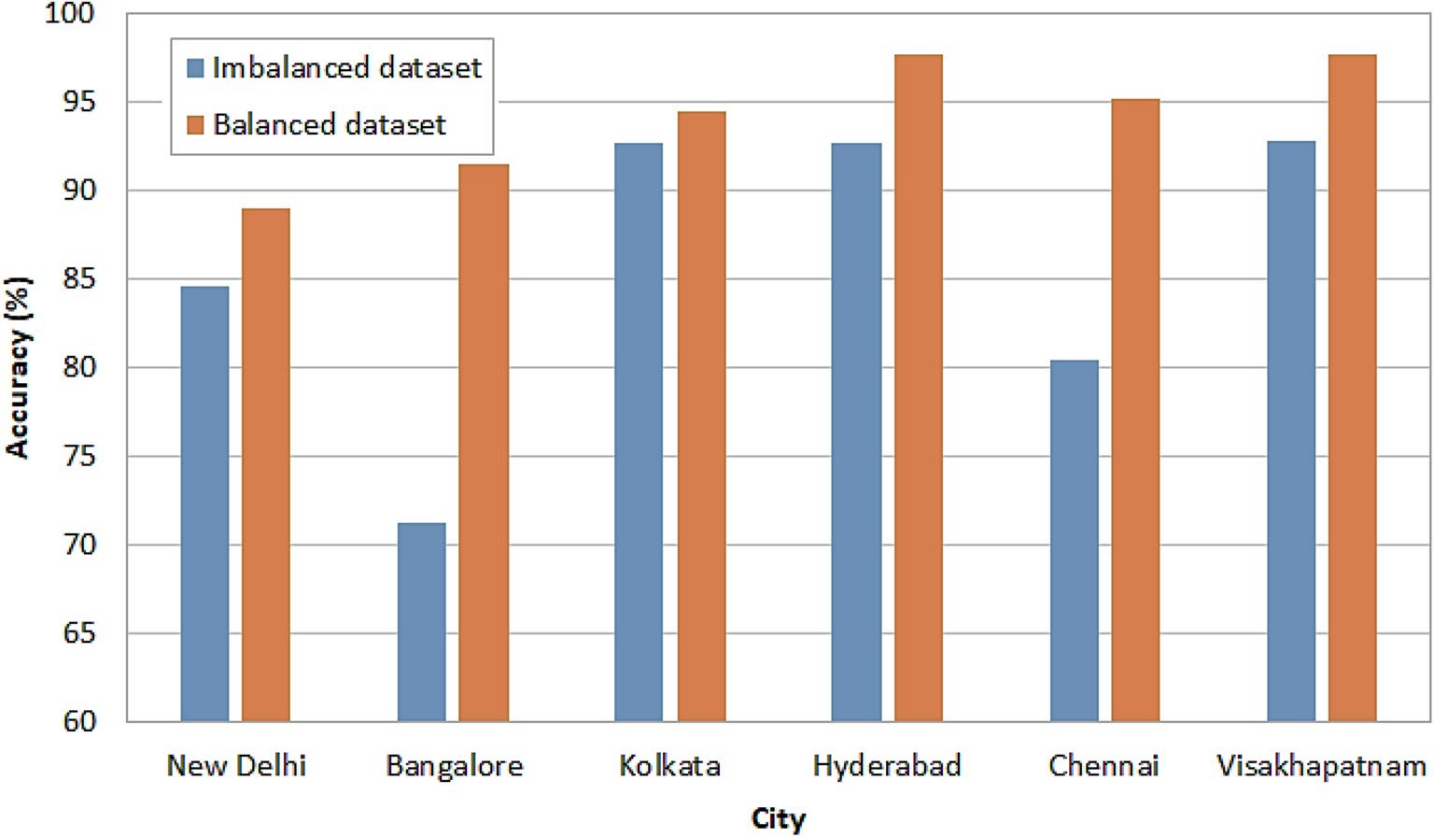
Accuracy analysis.

The accuracy analysis of proposed model for balanced and imbalanced dataset is presented in Fig. 16. It can be observed from the results that the balanced dataset attained better accuracy over imbalanced dataset. The accuracy attained for the New Delhi is 84.658% for imbalanced dataset, whereas for balanced dataset 88.98% was attained by the proposed model. The accuracy attained for the Bangalore city is 71.23% for imbalanced dataset, 91.49% for balanced dataset. The accuracy attained for the Kolkata city is 92.65% when using imbalanced dataset and 94.48% for the balanced dataset. The accuracy attained for the Hyderabad city is 92.68% for imbalanced dataset, 97.66% for balanced dataset. The accuracy attained for the Chennai city is 80.49% for imbalanced dataset, 95.22% for balanced dataset. The accuracy attained for the Visakhapatnam city is 92.84% for imbalanced dataset, 97.68% for balanced dataset.

### Comparative analysis

Further to compare the proposed model performance with conventional regression model like random forest regression, support vector regression, and k-nearest neighbor models. From the results of all models, it can be summarized that the proposed model attained maximum accuracy compared to conventional regression models.

The average accuracy attained by the proposed model for all the cities is 94.25% whereas support vector regression attained average accuracy of 90.34%, K-Nearest Neighbor attained average accuracy of 90.51% which is 4% lesser than the proposed model. The accuracy attained by the random forest regression is 92.75% which is 2% lesser than the proposed model. The comparison performance is shown in Table 8. Due to optimal feature selection, the decision tree regression model exhibited enhanced prediction performance and it can be seen from the results. From the result figures and tabulations, it can be observed that the proposed optimized regression model attained maximum performance over conventional technique.

**Table 8:** Performance comparison with conventional algorithms.

S. no.	City	Accuracy			
		Support vector regression	K-nearest neighbor	Random forest regression	Proposed GWO-DT
1	New Delhi	84.83	83.68	84.73	88.98
2	Bangalore	87.18	89.47	90.31	91.49
3	Kolkata	91.56	90.65	93.74	94.48
4	Hyderabad	93.57	93.68	97.61	97.66
5	Chennai	92.65	93.48	94.48	95.22
6	Visakhapatnam	92.24	92.11	95.65	97.68

## Conclusion

An optimized machine learning model for predicting Air quality index (AQI) in major cities of India is presented in this research work. The proposed prediction model includes grey wolf optimization algorithm and decision tree regression model for predicting AQI for major cities in India. Using the optimization model the optimal features are extracted from the historical data and fed into regression model for prediction process. Benchmark air quality data is used for the proposed model evaluation and major cities like New Delhi, Kolkata, Hyderabad, Chennai, Bangalore, and Visakhapatnam are considered for analysis. The prediction performance of proposed model is evaluated for the major cities using mean square error, mean absolute error, root mean square error, and accuracy metrics. Proposed model exhibited its maximum accuracy compared to existing methods like support vector regression, k-nearest neighbor, random forest regression models. With an maximum accuracy of of 88.98% for New Delhi city, 91.49% for Bangalore city, 94.48% for Kolkata, 97.66% for Hyderabad, 95.22% for Chennai and 97.68% for Visakhapatnam city experimentations confirmed the better performance of proposed model. In future the proposed model can be extended using deep learning models for attaining better prediction performances in air quality monitoring.

## Data Availability

The datasets used and/or analysed during the current study are available from the corresponding author on request.
